# Protective Immunity Induced by DNA Vaccination against Ranavirus Infection in Chinese Giant Salamander *Andrias davidianus*

**DOI:** 10.3390/v10020052

**Published:** 2018-01-24

**Authors:** Zhong-Yuan Chen, Tao Li, Xiao-Chan Gao, Chen-Fei Wang, Qi-Ya Zhang

**Affiliations:** 1 State Key Laboratory of Freshwater Ecology and Biotechnology, Institute of Hydrobiology, Chinese Academy of Sciences, Wuhan 430072, China; chenzy@ihb.ac.cn (Z.-Y.C.); lit@ihb.ac.cn (T.L.); gaoxiaochansx@163.com (X.-C.G.); 2 Wang’s Giant Salamander Breeding Professional Cooperative, Shiyan 442013, China; m17720267077@163.com

**Keywords:** ranavirus, *Andrias davidianus* ranavirus, Chinese giant salamander, DNA vaccine, protective immunity

## Abstract

*Andrias davidianus* ranavirus (ADRV) is an emerging viral pathogen that causes severe systemic hemorrhagic disease in Chinese giant salamanders. There is an urgent need for developing an effective vaccine against this fatal disease. In this study, DNA vaccines containing the ADRV *2L* gene (pcDNA-2L) and the *58L* gene (pcDNA-58L) were respectively constructed, and their immune protective effects were evaluated in Chinese giant salamanders. In vitro and in vivo expression of the vaccine plasmids were confirmed in transfected cells and muscle tissues of vaccinated Chinese giant salamanders by using immunoblot analysis or RT-PCR. Following ADRV challenge, the Chinese giant salamanders vaccinated with pcDNA-2L showed a relative percent survival (RPS) of 66.7%, which was significant higher than that in Chinese giant salamanders immunized with pcDNA-58L (RPS of 3.3%). Moreover, the specific antibody against ADRV was detected in Chinese giant salamanders vaccinated with pcDNA-2L at 14 and 21 days post-vaccination by indirect enzyme-linked immunosorbent assay (ELISA). Transcriptional analysis revealed that the expression levels of immune-related genes including type I interferon (*IFN*), myxovirus resistance (*Mx*), major histocompatibility complex class IA (*MHC* *IA*), and immunoglobulin M (*IgM*) were strongly up-regulated after vaccination with pcDNA-2L. Furthermore, vaccination with pcDNA-2L significantly suppressed the virus replication, which was seen by a low viral load in the spleen of Chinese giant salamander survivals after ADRV challenge. These results indicated that pcDNA-2L could induce a significant innate immune response and an adaptive immune response involving both humoral and cell-mediated immunity that conferred effective protection against ADRV infection, and might be a potential vaccine candidate for controlling ADRV disease in Chinese giant salamanders.

## 1. Introduction

Ranaviruses (genus *Ranavirus*, family *Iridoviridae*) are large, double-stranded DNA (dsDNA) viruses that infect a wide range of ectothermic vertebrates including amphibians, reptiles, and fish [1]. Ranaviruses are often associated with epidemics and mass die-offs of amphibian species worldwide and have been recognized as emerging infectious pathogens that pose a significant threat to global amphibian populations and biodiversity [2,3,4]. The Chinese giant salamander *Andrias davidianus* is the largest extant amphibian species and is a critically endangered species in China. In view of its high medicinal, nutritional, and scientific value, the Chinese giant salamander has been artificially farmed in many locations throughout China [5]. Unfortunately, viral epidemic diseases, especially ranavirus infections, have increasingly occurred in farmed Chinese giant salamanders since 2010, which have caused major impacts to Chinese giant salamander cultures and resulted in a great threat to the conservation of wild Chinese giant salamanders [6,7,8]. A lethal ranavirus, *Andrias davidianus* ranavirus (ADRV), was recently isolated and identified from Chinese giant salamanders [9]. ADRV infection caused a serious systemic hemorrhagic disease and led to more than 90% mortality in Chinese giant salamanders [6,7,9]. Although significant progresses have been achieved in understanding the pathogenesis and genome structure of ADRV as well as host immune responses against viral infection [9,10,11,12,13,14,15], no effective treatment for the virus is available. 

Vaccination has been considered one of the most effective approaches for controlling viral diseases in aquatic animals. For ranavirus diseases of Chinese giant salamanders, several candidate vaccines, such as an inactivated virus vaccine, a virus-like particle vaccine, and a recombinant subunit vaccine, have been developed. These vaccines conferred effective protection with a relative percentage survival of 72–84% against viral infection in laboratory trials [16,17,18]. DNA vaccines represent an attractive alternative to traditional vaccines, and they provide both humoral and cellular immunity and have various advantages including low production cost, high safety, and ease of long-term storage [19]. In aquaculture, DNA vaccines have been widely studied and are proven to be effective against viral pathogens such as the red seabream iridovirus (RSIV) [20], Singapore grouper iridovirus (SGIV) [21], and infectious spleen and kidney necrosis virus (ISKNV) [22].

Viral surface antigens are mainly localized to the outer envelope or capsid surface of virions [23,24]. Recently, *Rana grylio* virus (RGV) *2L* and *53R* were revealed to encode the viral envelope proteins, and the knockdown of *2L* and *53R* genes by artificial miRNAs or recombinant virus technology in RGV demonstrated that they play important roles in virus infection and assembly [25,26,27]. These features made them potential vaccine targets against viral infection. Analysis of the ADRV genome showed that ADRV contained 101 open reading frames (ORFs), among which ORF 2L and ORF 58L shared high homology with RGV 2L and 53R, respectively. In this study, we constructed DNA vaccines based on ADRV *2L* and *58L* genes and evaluated their protective potential and immunological effects in Chinese giant salamanders. 

## 2. Materials and Methods

### 2.1. Ethics Statement

All animal procedures were performed according to the recommendations in the Regulations for the Administration of Affairs Concerning Experimental Animals of China. The protocol was approved by the Institutional Animal Care and Use Committee of the Institute of Hydrobiology, Chinese Academy of Sciences (Y51317-1-301, 15 April 2015). All efforts were made to minimize suffering.

### 2.2. Chinese Giant Salamanders, Cells, and Virus

Healthy Chinese giant salamanders (about one year old and weighing 126.1 ± 15.6 g) were obtained from the farm of Wang’s Giant Salamander Breeding Professional Cooperative in Shiyan, Hubei Province, China. The Chinese giant salamanders were maintained in aerated freshwater tanks at 20–22 °C and were fed daily with silver carp. Prior to the experiments, the Chinese giant salamanders were acclimatized to laboratory conditions for two weeks and were assured free of ADRV infections by PCR assay. *Epithelioma papulosum* cyprinid (EPC) cells were cultured at 20 °C in medium 199 with 10% fetal bovine serum (FBS). ADRV was originally isolated in our laboratory and propagated in EPC cells as described previously [9].

### 2.3. Plasmid Construction

The *2L* and *58L* genes of ADRV were respectively amplified from genomic DNA using specific primers 2L-F1/R1 and 58L-F1/R1 (see Table S1). The obtained fragments were cloned into eukaryotic expression vector pcDNA3.1 (Invitrogen, Carlsbad, CA, USA) by corresponding restriction enzymes respectively. The recombinant plasmids, designated as pcDNA-2L and pcDNA-58L, was confirmed by using restriction enzyme digestion and DNA sequencing. Both plasmids (pcDNA-2L and pcDNA-58L) and the empty vector (pcDNA3.1) were purified from overnight cultures of transformed *Escherichia coli* strain DH5α cells with using the Endo-free Plasmid Midi Kit (Omega Bio-Tek, Norcross, GA, USA). 

### 2.4. Detection of Expression of Vaccine Plasmids in EPC Cells

EPC cells were seeded into 6-well culture plates and grown in medium199 supplemented with 5% fetal bovine serum (FBS). The cells were transfected with pcDNA-2L, pcDNA-58L, or the empty vector pcDNA3.1 using Lipofectamine^®^ 3000 (Invitrogen) according to the manufacturer’s instruction. Forty-eight hours after transfection, cells were harvested and lysed in sample loading buffer. Cell lysates were separated using 12% sodium dodecyl sulfate-polyacrylamide gel electrophoresis (SDS-PAGE) and transferred onto a polyvinylidene difluoride (PVDF) membrane for Western blot analysis, according to a previous report [28]. Mouse anti-ADRV 2L and anti-ADRV 58L sera (developed in the Laboratory of Aquatic Virology, Institute of Hydrobiology, Chinese Academy of Sciences, Wuhan, China) were used as primary antibodies, and alkaline phosphatase-conjugated goat anti-mouse IgG (Vector Laboratories, Youngstown, OH, USA) was used as a secondary antibody. The reactions were developed using substrate nitroblue tetrazolium (NBT) and 5-bromo-4-chloro-3-indolyl phosphate (BCIP) (Sigma, St. Louis, MO, USA). 

### 2.5. Vaccination and Challenge Experiment

The vaccine plasmids were diluted in sterile phosphate-buffered saline (PBS) to 400 μg/mL. The 120 healthy Chinese giant salamanders were randomly divided into three groups (40 animals/group). Animals were intramuscularly injected with 50 μL of 20 μg pcDNA-2L, pcDNA-58L, and pcDNA3.1, respectively, designated as the pcDNA-2L group, the pcDNA-58L group, and the pcDNA3.1 control group. At 21 days after vaccination, the Chinese giant salamanders from each group (30 animals/group) were intraperitoneally injected with 5 × 10^6^ TCID_50_ ADRV. Each group of Chinese giant salamanders was randomly distributed into three separate tanks. Mortality rates were recorded for 21 days after viral infection, and dead animals were removed daily. Relative percentage survival (RPS) was calculated according to the following formula [29]: RPS = [1 − (% mortality of vaccinated group/% mortality of control group)] × 100.

### 2.6. Detection of Expression of Vaccine Plasmids in Chinese Giant Salamanders

Muscle tissues at the injection site were taken from Chinese giant salamanders at seven days after vaccination, frozen in liquid nitrogen, and stored at −80 °C until used. Total RNA was extracted using TRIzol reagent (Invitrogen) based on the manufacturer’s instructions. Two micrograms of RNA were then reverse transcribed into cDNA using the PrimScript™ RT reagent Kit with gDNA Eraser (Takara, Tokyo, Japan). PCR was carried out with specific primers 2L-F2/R2 and 58L-F2/R2 (see Table S1). The *β-actin* gene was used as an internal control.

Total protein lysates were prepared through the homogenization of frozen muscle tissues in 50 mM Tris-HCl buffer (pH 7.5) with a protease inhibitor cocktail (Roche, Indianapolis, IN, USA). Lysates were centrifuged at 12,000× *g* for 20 min and the pellets were discarded. Samples were blotted onto the polyvinylidene difluoride (PVDF) membrane for immunodot blot. Mouse anti-ADRV 2L and anti-ADRV 58L sera were used as primary antibodies, and alkaline phosphatase-conjugated goat anti-mouse IgG (Vector Laboratories) was used as a secondary antibody. The blots were developed with NBT/BCIP (Sigma). ADRV suspension from infected EPC cell cultures was used for a positive control.

### 2.7. Antibody Enzyme-Linked Immunosorbent Assay (ELISA)

The presence of specific anti-ADRV immunoglobulin in vaccinated Chinese giant salamanders was examined by using indirect ELISA. Sera were collected from four animals of each treatment group, which were randomly selected, by tail venipuncture at 0, 7, 14, and 21 days after vaccination. The ELISA plates were coated with 1.0 μg per well of purified ADRV diluted in 100 μL of bicarbonate coating buffer (pH 9.6) at 4 °C overnight and then blocked with 5% bovine serum albumin (BSA) in PBS for 30 min at 37 °C. Diluted sera (1:40 dilution in PBS containing 1% BSA) were added in triplicate to the wells at 100 μL/well and incubated for 2 h at 37 °C. After washing three times with PBS containing 0.05% Tween-20 (PBST), mouse anti-Chinese giant salamander immunoglobulins polyclonal antibody (developed in the Laboratory of Aquatic Virology, Institute of Hydrobiology, Chinese Academy of Sciences, Wuhan, China) (1:2000 diluted in PBST) was added and incubated for 1 h at 37 °C. Subsequently, the plate was washed and further incubated with horseradish peroxidase conjugated goat anti-mouse IgG antibody (Vector Laboratories) (1:2000 diluted in PBST) for 1 h at 37 °C. After washing, substrate solution (0.1 M citrate/phosphate buffer, pH 5.0; 0.04% o-phenylenediamine; 0.14% H_2_O_2_) was applied for 10 min at room temperature. Reactions were stopped by adding 50 μL per well 2 M H_2_SO_4_, and optical densities (ODs) were measured at 492 nm with a microplate reader (Bio-Rad, Hercules, CA, USA).

### 2.8. Quantitative Real-Time PCR (qRT-PCR) Analysis of Expression of Immune Genes

The liver, spleen and kidney tissues were taken from three Chinese giant salamanders in each group at seven days after vaccination. Total RNA extraction and cDNA synthesis were performed as described above. qRT-PCR was carried out using Fast SYBR Green Master Mix with the StepOne^TM^ Real-Time PCR System (Applied Biosystems, Foster City, CA, USA) as described previously [10]. The primers for Chinese giant salamander type I interferon (*IFN*), myxovirus resistance (*Mx*), major histocompatibility complex class IA (*MHC IA*), immunoglobulin M (*IgM*), and *β-actin* are listed in Table S1. All samples were tested in triplicate, and the relative expression levels of target genes were determined using *β-actin* as an internal control with the comparative Ct (2^−ΔΔ*C*t^) method [30].

### 2.9. Determination of ADRV Viral Load by qRT-PCR

Ten surviving individuals from the pcDNA-2L group and one survivor from the pcDNA-58L group at 35 days after infection and 10 dead individuals from pcDNA 3.1 group during the viral challenge were randomly selected to evaluate the ADRV viral load. DNA was extracted from the spleen tissues of the sampled animals using the TaKaRa MiniBEST Universal Genomic DNA Extraction Kit (Takara) according to the manufacturer’s instructions. Quantification of viral copy numbers were determined by qRT-PCR with specific primers MCP-F1/R1 (see Table S1) for ADRV major capsid protein (MCP) gene according to the method described previously [31]. The viral load in samples was calculated as the mean of the three replicates and expressed as viral DNA copies per microgram of total DNA from tissue. 

### 2.10. Statistical Analysis

All data were analyzed using SPSS 17.0 software (SPSS Inc., Chicago, IL, USA) and expressed as mean ± standard error (SE). Differences in survival were determined with Chi-square test, and differences in all other assays were analyzed using the Student’s *t* test. Values of *p* < 0.05 were considered significant.

## 3. Results

### 3.1. Expression of Plasmid Constructs In Vitro and In Vivo

**Figure 1:**
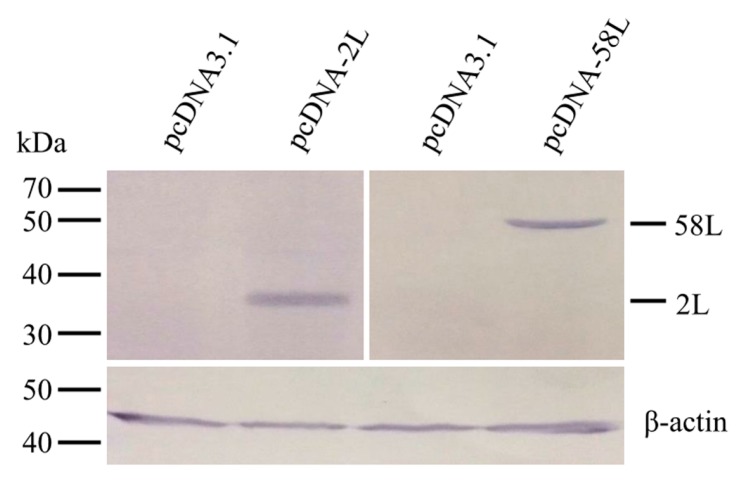
Expression of the 2L and 58L proteins in *Epithelioma papulosum* cyprinid (EPC) cells. EPC cells were transfected with pcDNA-2L, pcDNA-58L, or pcDNA3.1, and their expression were analyzed by Western blot at 48 h after transfection. The Chinese giant salamander β-actin was used as an internal control.

Expression of pcDNA-2L and pcDNA-58L in transfected EPC cells were confirmed by Western blot. As shown in Figure 1, the anti-ADRV 2L and anti-ADRV 58L antibodies specifically recognized the 35 kDa and 54.7 kDa protein bands from lysates of cells transfected with pcDNA-2L and pcDNA-58L, respectively. No specific protein band was detected in the lysates of pcDNA3.1 transfected cells.

**Figure 2:**
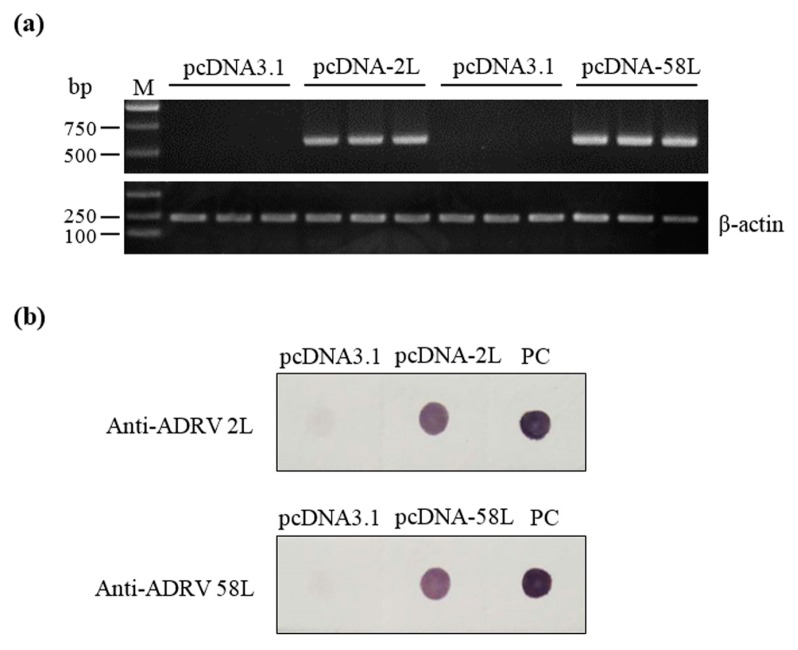
Expression of the 2L and 58L proteins in vaccinated Chinese giant salamanders. (**a**) RT-PCR analysis of the *2L* and *58L* gene transcriptions in muscle tissues from vaccinated Chinese giant salamanders at seven days post-vaccination (*n* = 3). The *β-actin* gene was used as an internal control. (**b**) Immunodot blot analysis of the expressed 2L and 58L proteins in muscle tissues from vaccinated Chinese giant salamanders at seven days post-vaccination. *Andrias davidianus* ranavirus (ADRV) suspension from infected EPC cell cultures was used for a positive control (PC).

RT-PCR and immunodot blot were performed to analyze the expression of pcDNA-2L and pcDNA-58L in the muscle tissues of Chinese giant salamanders at seven days post-vaccination. Transcripts of the *2L* and *58L* genes were detected in Chinese giant salamanders injected with pcDNA-2L and pcDNA-58L, respectively, while no amplification was obtained in negative control Chinese giant salamanders (see Figure 2a). Immunodot blot analysis showed that the lysates of muscle injected with pcDNA-2L and pcDNA-58L could be recognized by mouse anti-ADRV 2L and anti-ADRV 58L antibodies, respectively. No positive signal was observed in the lysates of muscle injected with pcDNA3.1 (see Figure 2b).

### 3.2. Specific Serum Antibody Response 

**Figure 3:**
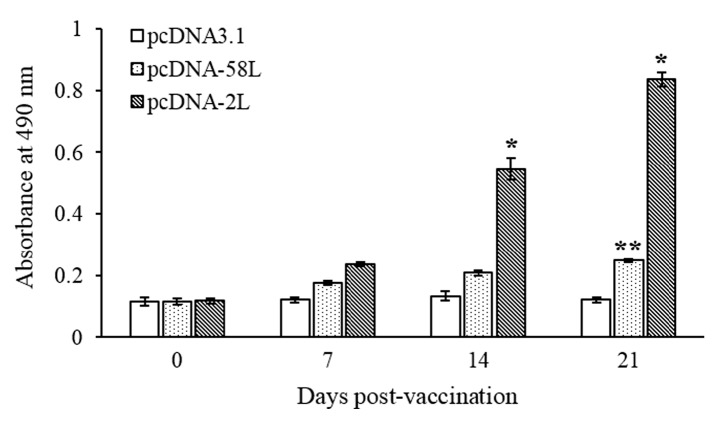
Serum antibody response in vaccinated Chinese giant salamanders. Sera were collected from Chinese giant salamanders at 0, 7, 14, and 21 days after vaccination, and serum antibodies against ADRV were determined by using indirect ELISA. Data are presented as means ± SE (*n* = 4). Asterisks indicate significant differences from the pcDNA3.1 group. * *p* < 0.05, ** *p* < 0.01.

The specific antibody in serum of vaccinated Chinese giant salamanders at different days post-vaccination were determined by using indirect ELISA. As shown in Figure 3, specific serum antibodies against ADRV were detected in Chinese giant salamanders of the pcDNA-2L group at 14 days post-vaccination, and the antibody level increased dramatically at 21 days post-vaccination. The Chinese giant salamanders of the pcDNA-58L group demonstrated a statistically significant increase (*p* < 0.01) in specific antibodies at 21 days post-vaccination compared to the control. No specific antibodies were detected in Chinese giant salamanders vaccinated with pcDNA3.1 (see Figure 3).

### 3.3. Expression of Immune-Related Genes

**Figure 4:**
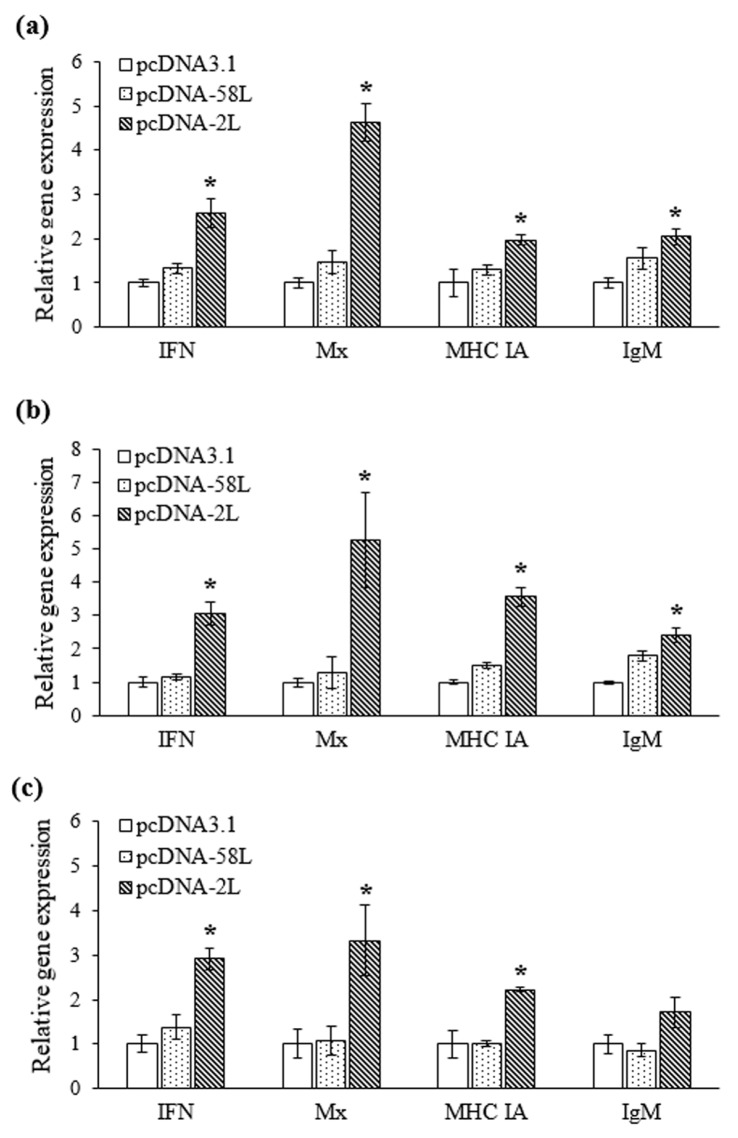
qRT-PCR analysis of the expression of type I *IFN*, *Mx*, *MHC IA*, and *IgM* genes in liver (**a**), spleen (**b**), and kidney (**c**) tissues from vaccinated Chinese giant salamanders. The mRNA level of each gene was normalized to that of *β-actin*. For each gene, the mRNA level of the control Chinese giant salamander (pcDNA3.1 group) was set as 1. Asterisks indicate significant differences from the control group. Data are presented as means ± SE (*n* = 3). * *p* < 0.05.

The expression levels of four immune-related genes were analyzed by using qRT-PCR in the liver, spleen and kidney from vaccinated Chinese giant salamanders at seven days post-vaccination. As shown in Figure 4, the expression of type I *IFN*, *Mx*, *MHC IA*, and *IgM* genes were differentially up-regulated in Chinese giant salamanders in the pcDNA-2L group compared with that in the pcDNA3.1 group. The transcription of type I *IFN* were significantly up-regulated more than 2.6-fold in the liver, spleen, and kidney, respectively. Meanwhile, expression of *Mx* was significantly increased with 4.6-, 5.3-, and 3.3-fold in the liver, spleen, and kidney, respectively. On the other hand, the mRNA levels of *MHC IA* were induced with up-regulation of 3.6-fold in the spleen, and no more than 2.2-fold in the liver and kidney, respectively. The expression of *IgM* was significantly up-regulated in the spleen and liver (2.4-fold and 2-fold, respectively), while a smaller increase (1.7-fold) was observed in the kidney. There was no significant difference in expression of all genes between pcDNA-58L and pcDNA3.1 groups. However, the expression of *IgM* showed a marginal increase (1.8-fold) in the spleen of Chinese giant salamanders vaccinated with pcDNA-58L. 

### 3.4. Protection of DNA Vaccination

**Figure 5:**
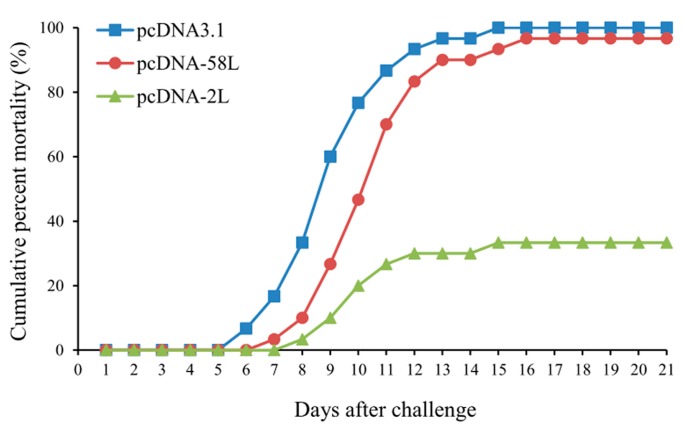
Cumulative mortality curves of vaccinated Chinese giant salamanders upon challenge with ADRV. The vaccinated Chinese giant salamanders were intraperitoneally injected with ADRV and monitored daily for mortality over a 21-day observation period.

To examine the protective effect of the vaccines, the vaccinated Chinese giant salamanders were challenged by intraperitoneal injection with ADRV at 21 days post-vaccination and monitored for mortality. As shown in Figure 5, the challenged Chinese giant salamanders began to die on days 6 in the pcDNA3.1 group after viral infection, followed on days 7 or 8 in the pcDNA-58L and pcDNA-2L groups. The cumulative mortalities reached 96.7% in the pcDNA-58L group and 33.3% in the pcDNA-2L group, whereas the cumulative mortalities of the pcDNA3.1 group was 100% (see Figure 5). Compared to the control group, the relative percent survival (RPS) values of the pcDNA-2L and pcDNA-58L groups were 66.7% and 3.3%, respectively (see Table 1). During the challenge trials, dead Chinese giant salamanders showed typical hemorrhage symptoms of ADRV infection and reisolation of ADRV by cultivating homogenates from the liver, spleen, and kidney tissues were positive for dying Chinese giant salamanders.

### 3.5. Detection of ADRV Viral Load 

The ADRV viral load in the spleen tissues of survivors from different vaccinated groups at 35 days after infection were further analyzed by using qRT-PCR. As shown in Table 2, the survivors from the pcDNA-2L group had a low level of viral load ranging from 1.00 × 10^1^ to 1.03 × 10^3^ copies/μg DNA, while the survivor from the pcDNA-58L group had a viral load of 1.75 × 10^3^ copies/μg DNA. In contrast, a high level of viral load with more than 1.42 × 10^7^ copies/μg DNA was detected in the spleen tissues of dead Chinese giant salamanders from the pcDNA 3.1 group during ADRV challenge. 

## 4. Discussion

Ranaviruses are known as emerging infectious pathogens with a broad host range and have been shown to impact amphibian population dynamics, which raised global concern [32,33,34]. As a rare and endangered amphibian species, the Chinese giant salamander has suffered from ranavirus diseases in natural habitats or on farms since 2010 [6,9,13]. In this study, two DNA vaccines (pcDNA-2L and pcDNA-58L) against ADRV were constructed, and the induced protective immunity was investigated in Chinese giant salamanders. To our knowledge, this is the first report of immune responses induced by DNA vaccination against viral infection in amphibians.

Virion structural proteins play a crucial role in the course of viral infections and are often involved in the induction of immune responses and antigen recognition [23]. ADRV *2L* and *58L* were revealed to encode the viral envelope proteins, and their homologues in other ranaviruses were proven to be essential for virus infection and assembly [25,27,35]. When used as antigen candidates to form a DNA vaccine in this study, immunologic analysis showed that pcDNA-2L effectively activated the immune system of vaccinated Chinese giant salamanders, whereas vaccination with pcDNA-58L generated, at best, a minimal response. Moreover, the Chinese giant salamanders vaccinated with pcDNA-2L exhibited significant protective effects against ADRV infection with an RPS of 66.7%, while vaccination with pcDNA-58L provided little, if any, protection (RPS of 3.3%). The results indicated the 2L protein could be an effective antigen candidate against ADRV. 

Active antibody responses are important for vaccine-mediated protection against viral diseases [36]. As demonstrated by the ELISA analysis, specific serum antibodies were detected in Chinese giant salamanders vaccinated with pcDNA-2L at 14 days post-vaccination, and the antibody level increased significantly at 21 days post-vaccination. The results were consistent with the observations in Chinese giant salamanders immunized with recombinant subunit vaccines based on the major capsid protein (a well-known immunogenic protein of the ranavirus) [17,18]. Similarly, adult *Xenopus laevis* infected with the ranavirus frog virus 3 (FV3) could generate specific anti-FV3 IgY antibodies, which neutralize the virus in vitro and provide partial passive protection to susceptible larvae [37]. It is plausible that pcDNA-2L can activate the specific humoral immunity in Chinese giant salamanders that may contribute to immune protection against ADRV.

It is well known that vaccination with an antigen can stimulate the expression of certain immune-related molecules or genes. In this study, transcriptional profiles of immune-related genes including type I *IFN*, *Mx*, *MHC IA*, and *IgM* were analyzed at seven days post-vaccination. As demonstrated, the expression of type I *IFN* and *Mx* genes were significantly up-regulated in liver, spleen, and kidney tissues in the pcDNA-2L group compared to the pcDNA-58L and control groups. Type I IFN plays a key role in innate antiviral immunity in vertebrates [38]. *Mx* is well known as type I IFN-inducible gene and the antiviral activities of Mx proteins have been extensively documented [39]. Recent report also revealed that the type I IFN of Chinese giant salamander could elicit significant increases in *Mx* gene expression and substantially reduce ranavirus replication and infection in vitro [40]. It is therefore tempting to speculate that pcDNA-2L triggered the type I IFN antiviral immune response in vaccinated Chinese giant salamanders, which was similar to the immune mechanism of DNA vaccines repelling viral pathogens in fish [19,41]. In addition, *MHC IA* expression was significantly up-regulated in the pcDNA-2L group. MHC class I molecules are responsible for binding foreign peptides when presenting them to cytotoxic T cells and have been recognized as significant elements of specific cell-mediated immunity in vertebrates [42]. The increased expression of *MHC IA* may indicate the recognition and presentation of pcDNA-2L performs in the MHC I pathway. Moreover, recent reports showed that the amphibian MHC I plays a potentially important role in conferring resistance to the ranavirus and MHC IA expression-deficient *Xenopus* tadpoles suffer from higher mortality rates than adults under ranavirus infection [43,44]. In accordance with specific antibodies detected in serum, the expression of *IgM* was significantly up-regulated in spleen and liver tissues within the pcDNA-2L group. Collectively, these results indicated that pcDNA-2L could activate the functions of the innate immune response as well as humoral and cell-mediated immune responses in Chinese giant salamanders. 

Previous reports have revealed that the spleen of Chinese giant salamanders is the main target organ of ADRV [9,13]. In this study, viral load in the spleen of Chinese giant salamander survivors at 35 days after ADRV infection was investigated. As demonstrated, the survivors in the pcDNA-2L group had a viral load of no more than 1.03 × 10^3^ copies/μg DNA, which was significantly lower than that of dead Chinese giant salamanders from the pcDNA 3.1 group during ADRV challenge. DNA vaccination trials in fish have showed that vaccinated fish do become infected following viral infection but are able to eliminate the infection and completely recover [19,28,41]. The low viral load in the survivors of pcDNA-2L group suggested that pcDNA-2L had triggered effective immune responses that resulted in inhibition of viral replication. However, the iridoviruses are often carried in the bodies of vertebrates, and many iridovirus infections may be chronic or conditional. As such, the infections are not easily inactivated in a host body [45,46]. It was also revealed that the ranavirus may exist long-term in asymptomatic Chinese giant salamanders [6,7]. The fate of ADRV in the survivors of vaccinated Chinese giant salamanders after infection needs further investigation. 

In conclusion, our results revealed that pcDNA-2L, a DNA vaccine encoding ADRV 2L protein, conferred effective protection upon ADRV challenge in Chinese giant salamanders. Moreover, pcDNA-2L could induce significant innate immune response and adaptive immune response involving both humoral and cell-mediated immunity, which are essential for combating ADRV infections. 

## Figures and Tables

**Table 1:** Cumulative mortality and relative percentage survival (RPS) of vaccinated Chinese giant salamanders infected with ADRV.

Vaccinated Groups	Cumulative Mortality (Death/Total)	RPS ^a^
pcDNA-2L	33.3% (10/30)	66.7 *
pcDNA-58L	96.7% (29/30)	3.3
pcDNA3.1	100% (30/30)	-

^a^ RPS = [1 − (% mortality of vaccinated group/% mortality of control group)] × 100. * Significant difference (*p* < 0.05) in survival between vaccinated Chinese giant salamanders and control Chinese giant salamanders (pcDNA 3.1 group).

**Table 2:** ADRV viral load determined by qRT-PCR in the spleen tissues from the surviving and dead individuals of vaccinated Chinese giant salamanders after viral challenge.

Vaccinated Groups	No. of Animal ^a^	Animal Status Death (D) or Survival (S)	Viral Load ^b^
pcDNA-2L	A1	S	1.00 × 10^1^
	A2	S	8.36 × 10^1^
	A3	S	1.71 × 10^1^
	A4	S	1.21 × 10^2^
	A5	S	1.83 × 10^2^
	A6	S	1.03 × 10^3^
	A7	S	1.32 × 10^1^
	A8	S	4.44 × 10^1^
	A9	S	3.28 × 10^2^
	A10	S	6.09 × 10^1^
pcDNA-58L	B1	S	1.75 × 10^3^
pcDNA 3.1	C1	D	6.26 × 10^7^
	C2	D	1.08 × 10^8^
	C3	D	1.89 × 10^7^
	C4	D	1.75 × 10^7^
	C5	D	1.54 × 10^8^
	C6	D	1.66 × 10^7^
	C7	D	1.42 × 10^7^
	C8	D	1.91 × 10^7^
	C9	D	2.55 × 10^7^
	C10	D	5.37 × 10^7^

^a^ A and B indicate the survivors from the pcDNA-2L and pcDNA-58L groups at 35 days after viral challenge; C indicate the dead individuals from the pcDNA 3.1 group at 6–15 days after viral challenge. ^b^ Copies of viral DNA per μg total DNA of tissue.

## Supplementary Materials

The following is available online at http://www.mdpi.com/1999-4915/10/2/52/s1, Table S1. Primers used in this study for plasmid construction, PCR detection, and gene expression analysis.
