# Supplementary material for: Protective Immunity Induced by DNA Vaccination against Ranavirus Infection in Chinese Giant Salamander Andrias davidianus
Source: Viruses. 2018 Jan 24;10(2):52. doi: 10.3390/v10020052 (PMC5850359; doi:10.3390/v10020052)
Supplement: Supplementary file 1 [file viruses-10-00052-s001.pdf]

Table S1 Primers used in this study for plasmid construction, PCR detection and gene expression analysis.

Primer name	Sequence (5′-3′)[a]	Usage
2L-F1	CTGGTACCATGTCCATCATCGGAG (*Kpn I*)	Plasmid construction
2L-R1	ATGAATTCTTACCATCTCACTGTAGA (*EcoR I*)	
58L-F1	TAGGATCCATGGGAGCAGCGGAA (*BamH I*)	
58L-R1	ATGAATTCTTAACCCCTGTGGGC (*EcoR I*)	
2L-F2	GAGCGACCAGATTACAGAACG	RT-PCR
2L-R2	ACTCGCCGAACATGGACACA	
58L-F2	GGCCTAAACCTGGCGCAATA	
58L-R2	ACCAGAACGATGATGACGATGA	
IFN-F	ATTGGCGTGCCTTTTCGTGCTATT	Real-time PCR
IFN-R	GGGAAAGTGTCCACCCATCTGCTC	
Mx-F	CATCCCTCGCCGACTTTGA	
Mx-R	CCAGAGAGTGCTTCAAGAACTGAG	
MHC IA-F	GGACTTCATCAGCCTCCACA	
MHC IA-R	AGGTTCCGTCAGGGTTCG	
IgM-F	TGCACGTGTATCACCCCCC	
IgM-R	AGCGTCCAGCAGTCCAGT	
MCP-F1	CAGTCAGGGACATGGTCGTG	
MCP-R1	GGGAGTGACGCAGGTGTAAT	
β-Actin-F	CCACTGCTGCCTCCTCTT	
β-Actin-R	GCAATGCCTGGGTACATG	

[a] Underlined nucleotides are restriction enzyme sites indicated in the brackets at the ends.
